# *Lactobacillus plantarum* and propionic acid improve the fermentation quality of high-moisture amaranth silage by altering the microbial community composition

**DOI:** 10.3389/fmicb.2022.1066641

**Published:** 2022-12-21

**Authors:** Muqier Zhao, Zhijun Wang, Shuai Du, Lin Sun, Jian Bao, Junfeng Hao, Gentu Ge

**Affiliations:** ^1^Key Laboratory of Forage Cultivation, Processing and High Efficient Utilization of Ministry of Agriculture and Rural Affairs, Inner Mongolia Agricultural University, Hohhot, China; ^2^Key Laboratory of Grassland Resources of Ministry of Education, Inner Mongolia Agricultural University, Hohhot, China; ^3^National Engineering Laboratory of Biological Feed Safety and Pollution Prevention and Control, Key Laboratory of Animal Nutrition and Feed Science of Zhejiang Province, Institute of Feed Science, Zhejiang University, Hangzhou, China; ^4^Inner Mongolia Academy of Agricultural and Animal Husbandry Sciences, Hohhot, China

**Keywords:** amaranth, fermentation quality, *Lactobacillus plantarum*, microbial community, propionic acid, silage

## Abstract

**Objective:**

The objective of this study was to determine the effect of *Lactobacillus plantarum* (*L. plantarum*) and propionic acid (PA) on the microbial community and fermentation performance of high-moisture amaranth silage.

**Methods:**

Amaranth silages were rown without addition (AhGCK) as a control and with *L. plantarum* JYLP-002 (AhGLP) or propionic acid (AhGPA) and then were opened after 60 days of ensiling to determine the microbial community and fermentation quality.

**Results:**

Crude protein (CP) content, lactic acid (LA) content, and lactic acid bacteria (LAB) counts were significantly higher in AhGLP and AhGPA compared with those in AhGCK (*p* < 0.05). In contrast, pH, acetic acid (AA) content, and yeast and aerobic bacteria counts were significantly lower in AhGLP and AhGPA compared with those in AhGCK (*p* < 0.05). In addition, propionic acid (PA) levels were markedly higher in AhGPA (*p* < 0.05). In terms of microbial communities, the silage in the additive groups showed an increased relative abundance of *Lactiplantibacillus plantarum* and *Lentilactobacillus buchneri* and a reduced relative abundance of *Enterobacter cloacae* and *Clostridium tyrobutyricum*. The abundance of *Xanthomonas oryzae* was significantly increased in AhGPA, but completely inhibited in the silage supplemented with *L. plantarum*. Spearman’s correlation analysis revealed that *Lentilactobacillus buchneri* and *Levilactobacillus brevis* were positively associated with LA and negatively associated with pH. Conversely, *Clostridium tyrobutyricum* and *Enterobacter cloacae* were negatively associated with LA, but positively associated with pH and AA content. AA content was inversely correlated with *Lentilactobacillus buchneri*. Functional prediction analysis showed that LAB dominated the three groups of silage and the silages containing additives had improved carbohydrate and amino acid metabolism compared with the control silage; in particular, the AhGLP group had more heterotypic fermentation processes and a richer metabolic pathway. Furthermore, the epiphytic *Lactiplantibacillus plantarum* and *Lentilactobacillus buchneri* could inhibit the reproductive activity of undesirable microorganisms to a certain extent, thus slowing the spoilage process of the silage.

**Conclusion:**

In conclusion, *L. plantarum* can improve fermentation characteristics by modulating the microbial community attached to high-moisture amaranth silage and will prove useful for preserving high-moisture silage.

## Introduction

Modern livestock systems have an enormous impact on the environment. Designing sustainable and intensive livestock production systems is a promising way to effectively reduce feed costs and achieve local access to local feed resources (Herrero et al., 2015). Silage storage technology is widely used to increase the productivity of animals and improve the efficiency of the livestock system when forage supplies are insufficient to maintain the productive performance of ruminants (Wilkinson et al., 2020). The main principle of silage fermentation is the production of lactic acid (LA) by fresh crop-attached lactic acid bacteria (LAB) under anaerobic conditions using water-soluble carbohydrates (WSC) as a fermentation substrate, which in turn creates an acidic environment and inhibits spoilage microorganisms (Dong et al., 2019). In China, the predominant limiting factor for livestock production is the shortage of fodder, which primarily occurs during the dry season. Consequently, Consequently, it is crucial that new, readily available, feed resources are developed to deal with such feed shortages (Du et al., 2021). Amaranth (*Amaranthus hypochondriaus*), an annual herb that has been cultivated for 8,000 years, may be a potential protein-feed resource. In recent years, some amaranth species have been used as forage crops owing their strong environmental adaptability (Rezaei et al., 2014; Peiretti, 2018). *A. hypochondriaus* is a C4 plant capable of producing 16.7 t/ha of dry matter (DM). In addition, it is one of the preferred feeds for sheep and beef and dairy cattle (Rahjerdi et al., 2015). Fresh grass yields of 86.4 t/ha and hay yields of 13.2 t/ha has been reported for amaranth (Abbasi et al., 2018). In addition, amaranth had a higher CP content (285 g/kg DM) and lower lignin content (40 g/kg DM) and lower nitrate and oxalate acid contents compared with those of maize (Rezaei et al., 2009; Rahjerdi et al., 2015). However, the high moisture and protein content of amaranth make it prone to spoilage. Exploring effective ways of preserving high-moisture amaranth is therefore crucial to develop this plant for use as forage in the livestock industry.

Silage quality can be directly improved by the administration of organic acids. Previous studies have compared the effects of formic acid (FA) and propionic acid (PA) on maize silage, with PA additives retaining more nutrients in maize silage and being able to be sprayed onto the forage, thus proving more beneficial than FA (Gheller et al., 2021). The PA additive inhibits yeasts and molds and can improve the aerobic stability of silage without additives (Kung et al., 2003; Coblentz and Akins, 2021). *Lactobacillus* inoculants have also been widely used in the silage fermentation process to produce high-quality silage. The inoculants were classified as homogeneous and heterogeneous fermenters based on their fermentation characteristics (Muck et al., 2018). Homogeneous LAB, represented by *L. plantarum*, grow and multiply rapidly. In the early stages of fermentation, they quickly produce LA, which lowers the pH of the system. This swift formation of an acidic environment in the fermentation system inhibits the growth and reproduction of harmful micro-organisms and effectively maintains feed nutrition (Ranjit and Kung, 2000). Researchers have inoculated *L. plantarum* in whole crop corn silage and Moringa oleifera leaf silage (Wang Y. et al., 2018), and sugarcane silage (Rabelo et al., 2019) and found that sugarcane and Moringa oleifera leaves exhibited better fermentation was achieved without the addition of *L. plantarum*. However, *L. plantarum* was highly effective as an inoculant for a high moisture amaranth and straw mixed silage. Compared with mixed silage without additives, *L. plantarum* increased the relative abundance of *Lactobacillus*, decreased the relative abundance of *Weissella*, *Pediococcus* and *Lactococcus*, decreased pH, and acetic acid (AA) and NH_3_-N concentrations, and increased concentration of LA (Mu et al., 2020). All types of microbial communities and biochemical reactions are involved in the ensiling process with fermentation quality predominantly dependent on the microbial community and its dynamic succession and fermentation metabolites (Yang et al., 2019). Consequently, obtaining detailed knowledge of the microbial community in silage is crucial to improve the quality of silage, especially in conditions of high moisture. In recent years, methods have been developed that allow the analysis of microbial communities in a culture-independent manner to circumvent the limitations of traditional culture methods (Ercolini, 2004). Next-generation sequencing (NGS) technology was employed to explore silage bacterial communities in the current study.

We hypothesized that inoculation of *L. plantarum* and PA additives during silage preparation might alter the bacterial community and succession patterns and affect fermentation performance in high-moisture seed amaranth silage. The effect of *L. plantarum* and PA on silage was investigated in terms of chemical composition, fermentation characteristics and bacterial community in conjunction with NGS techniques.

## Materials and methods

### Experimental design and silage preparation

The test material was red-fruited amaranth (*Amaranthus hypochondriaus*), which was planted on June 9, 2021 at the forage trial site of Inner Mongolia Agricultural University (111°430 E, 40°480 N, altitude 1,056 m above sea level) Hohhot, Inner Mongolia. Three 1-m^2^ plots were selected for harvesting (mature stage) on August 10. The collected red fruit amaranth was placed on a clean plastic sheet and was cut into lengths of approximately 3 cm using a hand-held guillotine (Mode-8,200; Minghong Business, Shandong, China).

For inoculant preparation, *Lactobacillus plantarum* (*L. plantarum*) JYLP-002 was supplied by Shandong Zhongke Jiayi Biological Engineering Company (Shandong, China). PA was manufactured by Shanghai McLean Biochemical Technology Company (Shanghai, China). Three treatment groups were established with the prepared amaranth: (1) no additive control (AhGCK); (2) *L. plantarum* JYLP-002 (AhGLP), applied into the fresh forage at a rate of 1 × 10^9^ cfu *L. plantarum*/g fresh matter (FM); (3) PA (AhGPA), added at 4 g/kg. The two additives were dissolved in deionized water, and 10 mL of solution per kg of amaranth forage was applied using a manual sprayer. The control silage was sprayed with an equivalent volume of deionized water. The amaranth (500 g) were packed in polyethylene plastic bags (size: 300 mm × 400 mm; Shenyang Huasheng Plastic Packaging Products Co., Ltd., Shenyang, China), which were then vacuum sealed (DZ400/2D vacuum sealer; Wenzhou Dafeng Machinery Co., Ltd., Wenzhou, China) and ensiled at ambient temperature (24–30°C) for 60 days. Chemical and fermentation characteristics, microbial populations, and microbial community of the control (AhGCK), *L. plantarum* (AhGLP), and PA (AhGPA) silages were then measured.

### Chemical and fermentation characteristics analysis

Each sample of fresh amaranth silage was replicated. Dry matter (DM) content was measured following the method of Zhang et al. (2016). The crude protein (CP) content was measured according to the method of Patrica (1997), utilizing on a Kjeldahl nitrogen tester (Gehart Vapodest 50 s, Germany). Neutral detergent fiber (NDF) and acid detergent fiber (ADF) content were measured using an Ankom A2000i fiber analyzer (Ankom Technology, Macedon, NY, USA) and the method of Van Soest (Van Soest et al., 1991). The WSC content was measured following the method of Chen et al. (2015).

A sample of silage (10 g) was mixed with 90 g deionized water following the description of Cai (2004) and stored in a refrigerator at 4°C for 24 h. The leachate was filtered through four layers of gauze and filter paper and the pH, ammonia nitrogen (NH_3_-N), and organic acids of the leachate were measured. pH was measured using a glass electrode pH meter (Leici pH S-3C, Shanghai, China). LA, AA, PA, and butyric acid (BA) contents of the silage were determined by high-performance liquid chromatography (HPLC; Model: Waters e2695, Milford, CT, USA) according to the method of Cheng et al. (2021). The method of Broderick and Kang (1980) was used to determine ammoniacal nitrogen (NH_3_-N) concentrations. Microbial populations (LAB, yeasts, mold, anerobic bacteria, and coliform bacteria) in the FM were assessed as described in a previous report (You et al., 2021).

### Microbial community analysis

The bacterial community compositions of ensiled high-moisture amaranth fermented for 60 days were analyzed by using 16S rRNA gene sequencing. Total DNA was extracted from the amaranth silage samples according to Liu et al. (2019). The procedures of metagenomic DNA extraction and PCR amplification of the bacterial 16S ribosomal RNA gene were performed according to Guo et al. (2021). Briefly, DNA was amplified with primers 27F (5′-AGRGTTTGATYNTGGCTCAG-3′) and 1492R (5′-TASGGHTACCTTGTTASGACTT-3′). PCR conditions were an initial denaturation at 98°C for 2 min, 30 cycles of denaturation at 98°C for 30 s, annealing at 50°C for 30 s, and elongation at 72°C for 60 s, followed by a final extension at 72°C for 5 min. The PCR products were purified for sequencing and analysis. Each treatment was performed in triplicate. DNA was amplified with primers 27F (5′-AGRGTTTGATYNTGGCTCAG-3′) and 1492R (5′-TASGGHTACCTTGTTASGACTT-3′). PCR conditions were an initial denaturation at 98°C for 2 min, 30 cycles of denaturation at 98°C for 30 s, annealing at 50°C for 30 s, and elongation at 72°C for 60 s, followed by a final extension at 72°C for 5 min. The PCR products were purified for sequencing and analysis. Each treatment was performed in triplicate.

Next-generation sequencing (NGS) sequencing was performed by Biomarker Technologies (Beijing, China) on a Pacbio_SMRT platform (Pacbio Sequel II, CA, USA). Coverage of alpha-diversity indicators Chao1 and Good was calculated using QIIME v1.9.1 (Caporaso et al., 2010). Principal coordinate analysis (PCoA) was performed using the R program (version 3.2.5) on the basis of beta-diversity unweighted or weighted unifrac distances. Operational taxonomic units (OTUs) were classified using Ribosome Database Project (RDP) Classifier (version 2.2) against the SILVA (Release 128) 16S rRNA database with a minimum confidence cutoff of 0.7, and were then denominated at the phylum, genus, and species levels. Mothur (version v.1.30) software was used to evaluate the alpha-diversity indices (ACE, Chao 1, Simpson, and Shannon) of the samples. A heat map of correlation analyses was produced using a R-based statistics tool. LEfSe [Linear Discriminant Analysis (LDA) effect size] was able to find biomarkers that are statistically different between groups (Segata et al., 2011). It was performed using a free online platform.^1^ Phylogenetic Investigation of Communities by Reconstruction of Unobserved States II (PICRUSt2) software was used to predict microbial functions from the Kyoto Encyclopedia of Genes and Genomes (KEGG) database. The PICRUSt2 software was used to analyze functional differences between different samples or subgroups by annotating the characteristic sequences to be predicted with the species in the phylogenetic tree available in the software and using the IMG microbial genomic data to output functional information and thus extrapolate the functional gene composition of the samples (Ward et al., 2017).

### Statistical analysis

One-way analyses of variance (ANOVA) based on SAS’ General Linear Model (GLM) program (version 9.3; SAS Institute Inc., Cary, NC, United States) were conducted on fermentation and nutritional characteristics and microbial counts of fresh and silage amaranth. Effects were considered significant when *p* < 0.05. The graphs of microbial community data were created using the BMK Cloud online platform and GraphPad prism 8.

## Results and discussion

### Chemical and microbial composition of pre-ensiled amaranth

The chemical composition and microbial counts of the raw material are presented in Table 1. The raw material had a low DM content (21.00%) and a CP content of 6.94% DM, which was lower than that reported by Mu et al. (2020), where the CP content of fresh amaranth was 91.9 g/kg at 136 g/kg DM. The low DM content might affect silage production, as high moisture content previously led to nutrient losses and markedly increased clostridial fermentation (He et al., 2020b). The NDF and ADF contents of the feedstock were 46.37% DM and 33.22% DM, respectively. The WSC content was only 1.18% DM. This might be due to the fact that amaranth is a thermophilic plant and the amaranth variety in this study was grown in a different environment with a large diurnal temperature difference, which affected its growth and nutrient accumulation. The WSC content and epiphytic LAB count of the material are another two determinants of fermentation quality (Dong et al., 2020). Cai et al. (1999) indicated that a minimum of 10^5^ cfu/g of LAB was required for the storage of well-preserved silage. Zhang et al. (2016) suggested that the minimum WSC level for successful fermentation is 60 g/kg DM. The amount of LAB in the fresh material was 3.84 log_10_ cfu/g FM in the current study. Based on the criterion, fresh amaranth failed to meet the requirement. However, the high number of coliforms (2.73 log_10_ cfu/g FM), yeasts (4.55 log_10_ cfu/g FM), and molds (2.00 log_10_ cfu/g FM) in the fresh material were lower than the bacterial counts of harmful microorganisms attached to rice straw, *Leymus chinensis*, and paper mulberry ingredients by previous studies (He et al., 2020a; Wu B. et al., 2022; Wu C. et al., 2022). Thus, silage additives like LAB were subsequently found to be indispensable in the preparation of silage.

**TABLE 1:** Chemical and microbial composition of pre-ensiled amaranth.

Items	Raw material
DM (%DM)	21.00 ± 1.23
CP (%DM)	6.94 ± 0.06
NDF (%DM)	46.37 ± 2.68
ADF (%DM)	33.22 ± 1.05
WSC (%DM)	1.18 ± 0.05
Lactic acid bacteria (Log_10_ cfu/g FM)	3.84 ± 0.55
Coliform bacteria (Log_10_ cfu/g FM)	2.73 ± 2.53
Aerobic bacteria (Log_10_ cfu/g FM)	6.45 ± 0.73
Yeast (Log_10_ cfu/g FM)	4.55 ± 2.58
Mold (Log_10_ cfu/g FM)	2.00 ± 1.76

FM, fresh matter; DM, dry matter; CP, crude protein; NDF, neutral detergent fiber; ADF, acid detergent fiber; WSC, water-soluble carbohydrate.

### Chemical and fermentation characteristics and microbial populations after 60 days of ensiling

The chemical and fermentation characteristics and microbial populations of the silage at 60 days are shown in Table 2. A significantly higher CP content was found in AhGPA compared with that in AhGLP and AhGCK (*p* < 0.05). The addition of PA reduced the degradation of CP in silage and avoided nutrient losses during ensiling. The additive groups had lower levels of NDF and ADF compared with the AhGCK group (*p* > 0.05). The growth of objectionable microorganisms and the accumulation of NH_3_-N illustrated by Dong et al. (2020) were predominantly manifested by the low CP content and high NH_3_-N content of AhGCK, while the additive groups performed well. This was coincident with low pH inhibiting the growth and activity of undesirable microorganisms (Arriola et al., 2011; Heinritz et al., 2012).

**TABLE 2:** Chemical compositions, fermentation characteristics, and microbial populations on 60 days of ensiling.

Items	Treatment			SEM	*P*-value
	AhGCK	AhGLP	AhGPA		
DM (%DM)	20.90 ± 1.39^a^	21.13 ± 0.16^a^	21.03 ± 0.59^a^	0.07	0.9369
CP (%DM)	5.90 ± 0.69^b^	6.15 ± 0.26^b^	8.08 ± 0.12^a^	0.69	0.0060
NDF (%DM)	48.31 ± 2.19^a^	44.83 ± 2.69^a^	46.38 ± 2.41^a^	1.01	0.4983
ADF (%DM)	39.37 ± 1.18^a^	36.09 ± 1.58^a^	38.07 ± 2.15^a^	0.95	0.5142
WSC (%DM)	0.37 ± 0.10^a^	0.31 ± 0.09^a^	0.23 ± 0.05^a^	0.04	0.2476
pH	5.09 ± 0.39^a^	4.16 ± 0.05^b^	4.28 ± 0.12^b^	0.29	0.0111
Lactic acid (%DM)	1.42 ± 0.20^c^	3.65 ± 0.12^a^	2.52 ± 0.28^b^	0.65	< 0.0001
Acetic acid (%DM)	0.67 ± 0.10^a^	0.40 ± 0.09^b^	0.17 ± 0.04^c^	0.14	0.0034
Propionic acid (%DM)	0.35 ± 0.13^b^	0.12 ± 0.02^c^	0.98 ± 0.13^a^	0.26	0.0006
Butyric acid (%DM)	0.00	0.00	0.00	0.00	0.0000
NH_3_-N/TN (%)	1.42 ± 0.32^a^	1.02 ± 0.03^a^	1.22 ± 0.10^a^	0.12	0.2077
Lactic acid bacteria (Log_10_ cfu/g FM)	4.39 ± 0.27^a^	5.41 ± 0.79^a^	4.63 ± 0.40^a^	0.31	0.0494
Mold (Log_10_ cfu/g FM)	0.00^a^	0.00^a^	0.90 ± 1.56^a^	0.30	0.4444
Yeast (Log_10_ cfu/g FM)	7.49 ± 0.20^a^	5.39 ± 1.45^b^	4.37 ± 0.81^b^	0.92	0.0459
Anerobic bacteria (Log_10_ cfu/g FM)	7.76 ± 0.33^a^	4.32 ± 0.33^b^	4.04 ± 0.07^b^	1.20	0.0002
Coliform bacteria (Log_10_ cfu/g FM)	0.00	0.00	0.00	0.00	0.0000

Different lowercase letters indicate significant differences among different treatments (*p* < 0.05); same letter indicate not significant (*p* > 0.05).

Compared with the control silage, the inoculated silage had a lower WSC content, which was congruent with the findings of Kleinschmit and Kung (2006). During the fermentation process, in response to LAB fermentation, WSC is converted to organic acids, ethanol, and carbon dioxide (Muck et al., 2018; Wang S. et al., 2018). A high moisture content was previously reported to be detrimental to natural fermentation and led to a decrease in organic acid concentration and an increase in pH, which adversely affected the fermentation process of alfalfa silage (Cai et al., 1998; Zheng et al., 2017). Both AhGLP and AhGPA had a significantly lower pH compared with AhGCK (*p* < 0.05); in particular, the pH of AhGLP was lower than 4.20. These results were congruent with the conclusions of Wang et al. (2019), who considered that LAB accelerated the accumulation of LA and lowered the pH of silage. Yang et al. (2019) also concluded that the growth of harmful bacteria was effectively inhibited when the pH was below 4.20, ensuring quality fermentation, and that the addition of LAB accelerated the LA fermentation process of alfalfa silage. In contrast, the pH of AhGCK in our study was as high as 5.09. This can potentially be attributed to the high metabolism of LA and other nutrients consumed by yeasts, molds, and other aerobic microorganisms, which results in a rise in pH (Woolford, 1990). *Escherichia coli* and molds were undetectable after 60 days of fermentation in high-moisture amaranth silages, congruent with the report of Dong et al. (2020). This was due to the low pH suppressing the activities of adverse microorganisms (Lima et al., 2010; Reich and Kung, 2010).

*Lactobacillus plantarum* JYLP-002 (AhGLP) had significantly higher LA content compared with AhGCK and AhGPA, but AhGPA had significantly lower and higher AA and PA contents, respectively, compared with AhGLP and AhGCK (*p* < 0.05). PA and BA are indicators of silage quality and consume some of the metabolic energy during production. It was previously reported that conversion of LA to BA resulted in the loss of more than half of the DM content and some total energy (Muck, 2010), and this impacted the feed intake of livestock (Dong et al., 2020). The BA content of the different silages in the present study was 0.00. Some microorganisms that were detrimental to silage fermentation (for example, yeast, molds, *Clostridium*, and *Enterobacter*) often attached to silage or poor quality silage at different times of fermentation (Driehuis et al., 2018; Zhang et al., 2019; Guo et al., 2020). As fermentation proceeds, these microorganisms were gradually inactivated or disappear. In silage, *Bacillus*, *Clostridium*, and *Enterobacter* and yeasts are the microorganisms that play a central role in BA formation by excreting amino acid decarboxylases to produce BA (Jia et al., 2021). The low abundance of these harmful microorganisms in this study may also be one of the reasons why BA was not detected. AhGPA had a higher PA content compared with AhGLP and AhGCK, and this might be attributed to the increase in exogenously added PA and silage fermentation products. Fermentation characteristics were positively affected by inoculation with *L. plantarum*; this LAB not only decreased the pH but also promoted the accumulation of LA.

### Bacterial community of high-moisture amaranth silages

The third-generation Pacific Biosciences (PacBio) SMRT has proved outstanding in its ability to boost the sensitivity and accuracy of microbial community classification in ensiling (Xu et al., 2019). Consequently, the bacterial communities of high-moisture amaranth silage in the current study were further investigated by sequencing using SMRT.

Table 3 shows the bacterial alpha diversity of the amaranth silages. Ensiling is a bacteria-driven process, and the type of bacteria and their abundance in the fermentation process can directly affect the quality of the fermentation (Guan et al., 2018; He et al., 2020a). Sequencing coverage values for all samples were greater than 99%, indicating that the sequencing depth was sufficient for effective bacterial community characterization. Alpha diversity reflects bacterial diversity and species richness in individual samples. Shannon and Simpson indices indicate species diversity, while Chao1 and ACE indices represent species richness. The Simpson index of AhGCK was higher compared with those of AhGLP and AhGPA, but not by a significant margin (*p* > 0.05). The two additive groups of silage had higher OTU, ACE, Chao1, and Shannon indices compared with those of AhGCK. Among them, the ACE and Chao1 indices were higher in AhGLP compared with those in AhGCK. These results were in accordance with the findings of Du et al. (2021) who detected an increase in Chao1 and Ace indices in paper mulberry silage after inoculation with LAB compared with the control group. This suggests that the anaerobic and acidic environment displaces a large number of aerobic microorganisms in the silage by beneficial microorganisms (e.g., LAB), which in turn affects the overall micro-ecological environment. Collectively, the above results indicate that the screened LAB in the current study can rapidly reduce the pH of amaranth silage, thereby inhibiting harmful microorganisms and diminishing the alpha diversity of bacteria (Li et al., 2021). Once LAB became the prevailing species, bacterial diversity decreased, which was similar to the observations of Ni et al. (2017). Fermentation with exogenous *L. plantarum* and PA positively impacted the production of LA. The results of current study further demonstrated that exogenous *L. plantarum* exhibits a strong competitive advantage during the fermentation of high-moisture amaranth silage. Therefore, there is a need to study the dominant species of bacterial communities in amaranth silage.

**TABLE 3:** Alpha-diversity of bacterial diversity of amaranth silages.

Items	Treatment			SEM	*P*-value
	AhGCK	AhGLP	AhGPA		
OTUs	45.0000^b^	112.5000^a^	95.5000^a^	20.27	0.0296
ACE	130.2034^a^	198.7408^a^	116.1056^a^	25.52	0.2751
Chao1	84.8301^b^	160.4670^a^	112.9263^ab^	22.07	0.1008
Simpson	0.6122^a^	0.5932^a^	0.5312^a^	0.02	0.7951
Shannon	1.8952^a^	2.3002^a^	2.2306^a^	0.13	0.7347
Coverage	0.9982^a^	0.9957^b^	0.9976^ab^	0.0008	0.0477

AhGCK, control; AhGLP, *L. plantarum*; AhGPA, propionic acid. Different lowercase letters indicate significant differences among different treatments (*p* < 0.05); same letter indicate not significant (*p* > 0.05).

**FIGURE 1:**
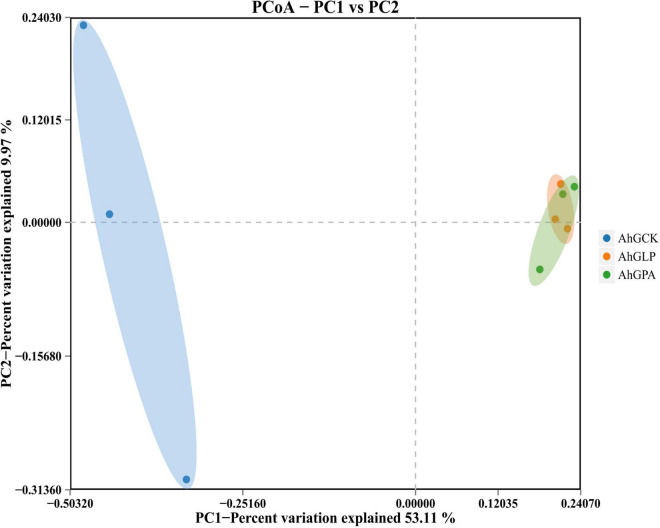
Bacterial β-diversity of high-moisture amaranth silage with *Lactobacillus plantarum* and propionic acid additives. AhGCK, control; AhGLP, *L. plantarum*; AhGPA, propionic acid.

Principal coordinate analysis (PCoA) clearly reflected the variations within the microbial community (Figure 1). There was a marked division of bacterial communities in the control and additive groups. The addition of additives highlighted the variability of bacterial communities in the silage samples of the different treatments, indicating that the composition and structure of the bacterial communities were significantly altered in AhGLP and AhGPA.

**FIGURE 2:**
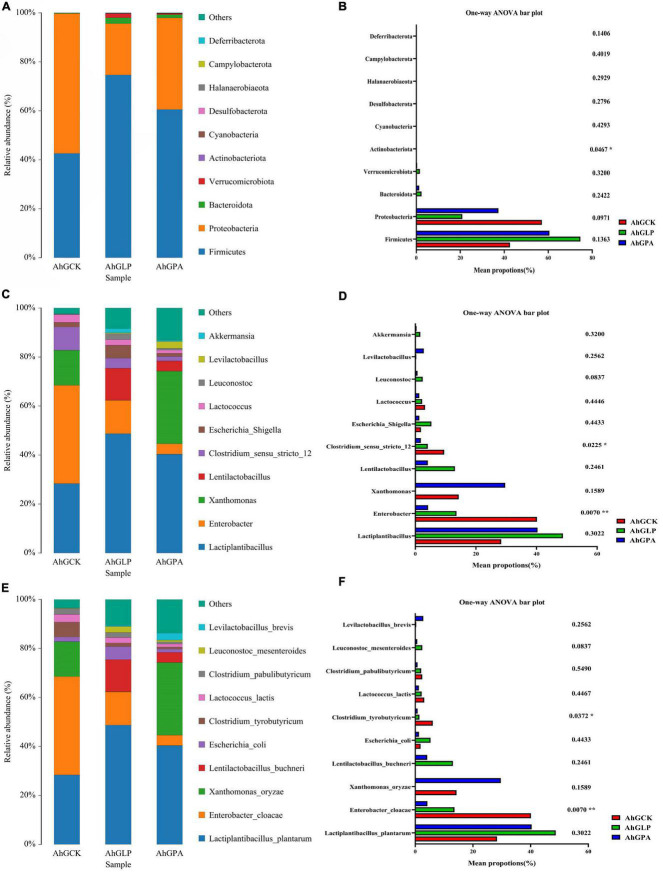
Relative abundance of bacteria at the phylum **(A)**, genus **(C)**, and species **(E)** levels in high-moisture amaranth silage with *Lactobacillus plantarum* and propionic acid additives. The extended error bar plot displaying the significant differences among groups at the phylum **(B)**, genus **(D)**, and species **(F)** levels. AhGCK, control; AhGLP, *L. plantarum*; AhGPA, propionic acid.

Of the numerous elements that influence the silage fermentation process, the predominant microbial species often determine the quality of the silage (Ennahar et al., 2003; Ni et al., 2017). Therefore, it is essential to analyze changes in fermentation characteristics and microbial composition during silage to facilitate understanding of the silage process and enhance fermentation quality (Namihira et al., 2010). The relative abundance of bacteria in amaranth forage is shown in Figure 2, with the bacterial community at the phylum level presented in Figure 2A. *Firmicutes*, *Proteobacteria*, and *Bacteroidota* were the dominant phyla in all three groups after 60 days of ensiling. There was a significant increase and decrease in the relative abundance of *Firmicutes* and *Proteobacteria*, respectively, in AhGLP and AhGPA compared with AhGCK. The relative abundance of *Firmicutes* was more than 60% in AhGLP and AhGPA, while the relative abundance of *Proteobacteria* was lower in these two groups compared with that in AhGCK. *Firmicutes* were the main clade in the silage in the present study, which was consistent with the findings of Bao et al. (2016). This might be due to the low pH or anaerobic conditions favoring the growth of members of the phylum *Firmicutes* during silage (Keshri et al., 2018). Figure 2B shows the significant differences among groups at the phylum level. It indicated that there were significant differences in *Actinobacteriota* between three groups (*p* < 0.05).

Relative abundances of bacteria at the genus level are presented in Figure 2C. The dominant genus in AhGCK was *Enterobacter*, and the subdominant genera were *Lactiplantibacillus* and *Xanthomonas*. The dominant genus in AhGLP and AhGPA was *Lactiplantibacillus*, while the subdominant genera in AhGLP were *Enterobacter*, *Lentilactobacillus*, and *Escherichia Shigella*, and those in AhGPA were *Enterobacter* and *Xanthomonas.* The relative abundances of *Lactobacillus* and *Enterobacter* in the additive groups were significantly higher and lower, respectively, compared with those in AhGCK, which could explain the high LAB populations in the additive groups. In a previous study, competition between *Enterobacter* and LAB for available WSC in silage led to the production of more AA, succinic acid, 2,3-butanediol, some endotoxins, and ammonia nitrogen and reduced nutritional value and palatability (Muck, 2010). Therefore, *Enterobacter* are an undesirable genus in silage. The dominant genus in AhGCK was *Enterobacter*, and this was confirmed by its poor silage quality. In AhGLP and AhGPA, *Enterobacter* was the second dominant genus after *Lactiplantibacillus*, but the addition of *L. plantarum* and PA reduced the abundance of *Enterobacter*. *Clostridium sensu stricto* 12 and *Enterobacter* had significant and highly significant differences among three groups (*p* < 0.01 and *p* < 0.05) (Figure 2D). We concluded that the intervention of additives in the silage fermentation process plays an important role in the fermentation of silage.

Figure 2E illustrates the relative abundance of the top 10 bacterial species in amaranth silage. LAB play an essential role in fermentation during the ensiling process. In this study, *Lactiplantibacillus plantarum*, *Enterobacter cloacae*, and *Xanthomonas oryzae* were the dominant species in AhGCK and AhGPA. PA is often used to improve the aerobic stability of silage. In the present study, the addition of PA enhanced the relative abundance of *Lactiplantibacillus plantarum* and *Lentibacillus buchneri*. Exogenous addition of PA lowers the pH owing to the acidity of the PA itself, thereby inhibiting acid-intolerant bacteria, creating a favorable anaerobic environment, encouraging acid-tolerant LAB fermentation (e.g., *Lactiplantibacillus plantarum* and *Lentibacillus buchneri*), and reducing undesirable fermentation and protein hydrolysis. Papendick and Singh-Verma (1972) and Jia et al. (2021) also produced similar results. The dominant species of AhGLP in the current study were *Lactiplantibacillus plantarum*, *Enterobacter cloacae*, and *Lentilactobacillus buchneri*, among which, *L. plantarum* is known to play a key role in silage fermentation. *Enterobacter cloacae* and *Clostridium tyrobutyricum* exhibited significant and highly significant differences among three groups (*p* < 0.01 and *p* < 0.05), and were the common harmful microorganisms found in high-moisture silage (Figure 2F). *Enterobacter cloacae, Clostridium tyrobutyricum*, and some yeasts produce biogenic amines (an endogenous metabolic component of cells in plants) by excreting amino acid decarboxylases (Halász et al., 1994). *X. oryzae* was the third-ranked dominant species in the silage of AhGCK and AhGPA. Members of the genus *Xanthomonas* are pathogenic for more than 300 plant species (Boch and Bonas, 2010; Büttner and Bonas, 2010). *X. oryzae* is obligatorily aerobic, does not form spores, grows optimally between 25 and 30°C, is peroxidase-positive, cannot reduce nitrate, and is a weak producer of acid from carbohydrates (Bradbury, 1984). *Xanthomonas* differs from non-pathogenic bacteria in that it relies on a variety of potent substances secreted through different kinds of protein secretion systems, predominantly the type III secretion system, to suppress host immunity and to obtain nutrients from plants. Amaranth was considered a promising crop in Argentina (Noelting et al., 2019), but was found to be a host of *Xanthomonas euvesicatoria* (Santos et al., 2020), *Curtobacterium flaccumfaciens* pv. *flaccumfaciens* (Nascimento et al., 2020), and *Xanthomonas citri* subsp. *citri* (Ebrahimi et al., 2020). It also demonstrates that *X. oryzae* accounts for a large proportion of amaranth silage. Our results show that *L. plantarum* might inhibit the growth and multiplication of pathogens. As a fermentation inhibitor, PA inhibited aerobic bacteria, mold, and *Bacillus* bacteria, but *X. oryzae* exhibited the opposite trend in the amaranth silage with PA (i.e., *X. oryzae* increased in abundance) compared with the amaranth silage with *L. plantarum* added (i.e., *X. oryzae* decreased in abundance). In addition, *X. oryzae* could not produce LA and AA with soluble sugar as the fermentation substrate, which also explains the poor fermentation quality in AhGPA compared with AhGLP. Thus, *L. plantarum* can affect the bacterial diversity and community structure of high moisture amaranth silage by inhibiting the colonization of undesirable microorganisms, such as *Enterobacter cloacae* and *Clostridium tyrobutyricum*.

**FIGURE 3:**
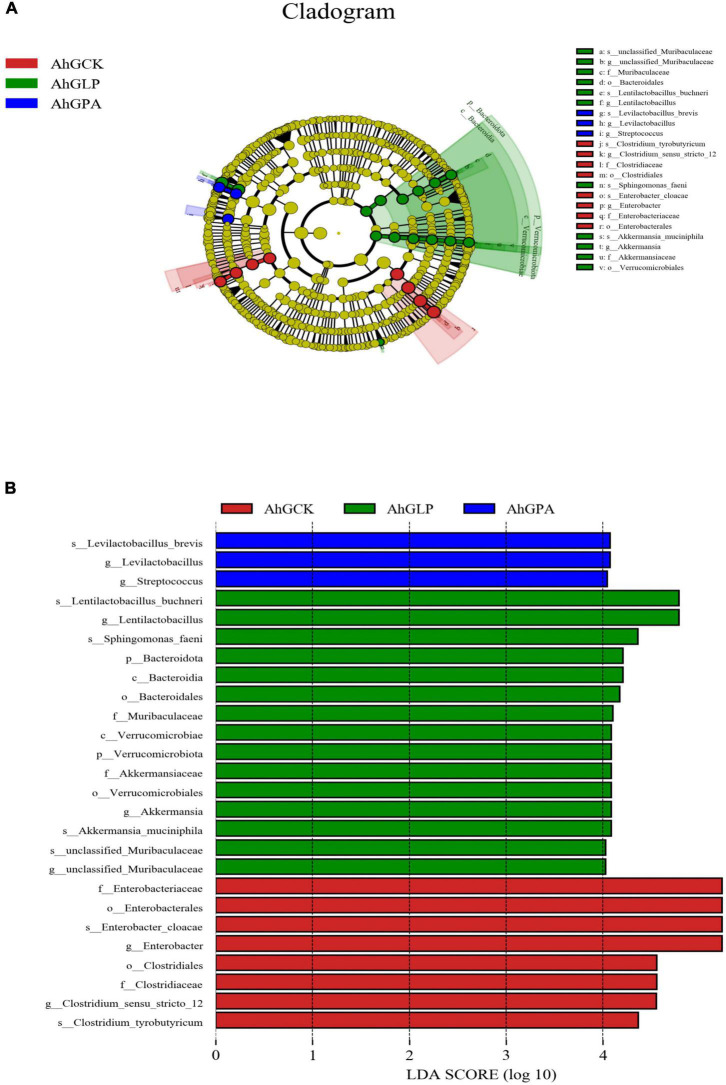
Comparison of microbial variations using the LEfSe online tool for high-moisture amaranth silage with *Lactobacillus plantarum* and propionic acid additives. AhGCK, control; AhGLP, *L. plantarum*; AhGPA, propionic acid. **(A)** Cladogram for taxonomic representation of significantly differences. **(B)** Indicator bacteria with an linear discriminant analysis (LDA) score of 4 or more in the silage bacterial community under different treatments.

The LEfSe method was used to evaluate differences in microbial communities between the three groups of silage and to explore specific bacterial species in each group [linear discriminant analysis (LDA) score, > 4.0]. *L. plantarum* markedly affected the microbial community in the silage (Figure 3). In AhGCK, a group of 8 bacteria were significantly enriched, with *Enterobacter cloacae* (LDA score, 5.60) having the highest LDA score. In AhGLP, a group of 11 bacteria were significantly enriched, with *Lentilactobacillus buchneri* (LDA score, 5.12) having the highest LDA score. In AhGPA, three subgroups of bacteria were significantly enriched, with *Levilactobacillus brevis* (LDA score, 4.42) having the highest LDA score. These results suggested that there was some variation in species abundance in specific communities of high-moisture amaranth silage with different additives. The reason of why the *Lactiplantibacillus plantarum* has no difference in the LEfSe after the addition of *Lactobacillus plantarum* may be due to the fact that *Lactiplantibacillus plantarum* was not the most dominant strain in all groups.

### Relationships between fermentation parameters and bacterial community

**FIGURE 4:**
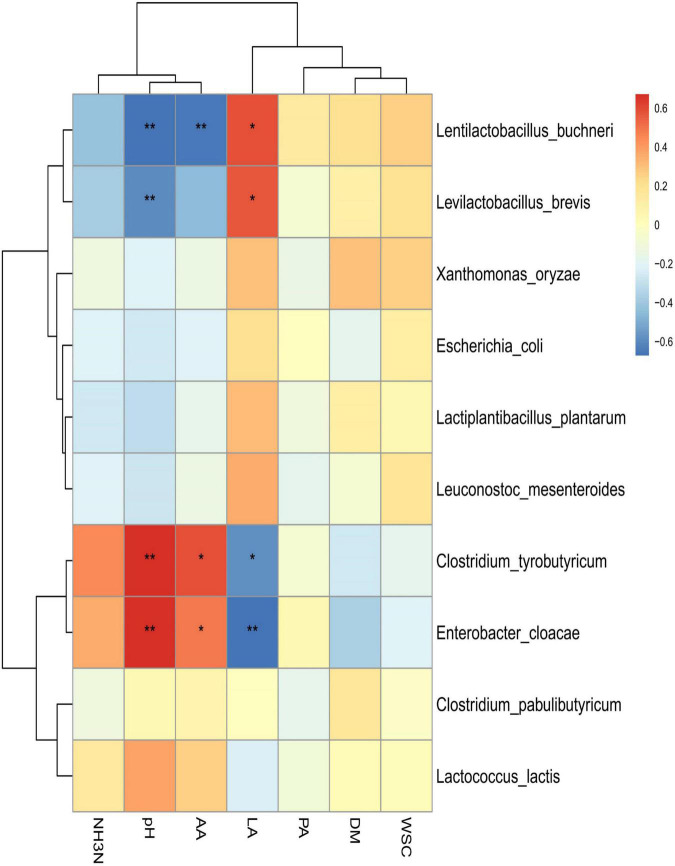
Spearman correlation heatmap of abundance of microbial community and fermentation properties in high-moisture amaranth silage with *Lactobacillus plantarum* and propionic acid additives. AhGCK, control; AhGLP, *L. plantarum*; AhGPA, propionic acid; AA, acetic acid; DM, dry matter; LA, lactic acid; PA, propionic acid; WSC, water soluble carbohydrate. *p < 0.05; **p < 0.01.

Spearman correlation clustering was used to explore correlation between microbial communities and fermentation characteristics (species level; Figure 4). *Lentilactobacillus buchneri* and *Levilactobacillus brevis* were positively associated with LA (*R*^2^ = 0.590 and *R*^2^ = 0.577, respectively; *p* < 0.05 for both), which suggested that the generation of LA was largely attributable to these two species. In addition, these two species were negatively associated with pH (*R*^2^ = –0.662 and *R*^2^ = –0.602 for *Lentilactobacillus buchneri* and *Levilactobacillus brevis*, respectively; *p* < 0.01 for both). *Clostridium tyrobutyricum* and *Enterobacter cloacae* were negatively associated with LA (*R*^2^ = –0.585, *p* < 0.05; *R*^2^ = –0.672, *p* < 0.01), but positively correlated with pH (*R*^2^ = 0.668 and *R*^2^ = 0.678, respectively; *p* < 0.05 for both) and AA (*R*^2^ = 0.589 and *R*^2^ = 0.485, respectively; *p* < 0.05 for both). AA was also inversely correlated with *Lentilactobacillus buchneri* (*R*^2^ = –0.654, *p* < 0.01).

*Lentilactobacillus buchneri* and *Levilactobacillus brevis* were previously found to be major producers of LA and were instrumental in lowering pH during ensiling (Cai et al., 1998; Wang et al., 2020; Yang et al., 2021). The low AA content indicated that heterofermentative LAB produce PA and AA through fermentation to enhance aerobic stability (Naiara et al., 2019). Fermentation products are the result of the joint action of the resident microorganisms, and eventually, more PA and a small amount of AA were produced during fermentation because ofthe different metabolic pathways of the microorganisms (Xu et al., 2019). Consequently, *Lentilactobacillus buchneri* may produce more products other than AA when fermenting through the HMP pathway (Muck et al., 2018). *Clostridium tyrobutyricum* and *Enterobacter cloacae* were the predominant contributors to the reduction in pH and increase in AA production and thus have a negative impact on the production of an acidic environment during the silage process. This may be the main reason for the growth and multiplication of undesirable bacteria attached to the silage and consequently increases nutrient losses.

In summary, this analysis demonstrated that *L. plantarum* and PA enhanced the fermentation characteristics of high-moisture amaranth by enhancing the relative abundance of *Lactiplantibacillus plantarum* and *Lentilactobacillus buchneri* and decreasing the relative abundance of *Enterobacter cloacae*.

### KEGG gene function predictions

**FIGURE 5:**
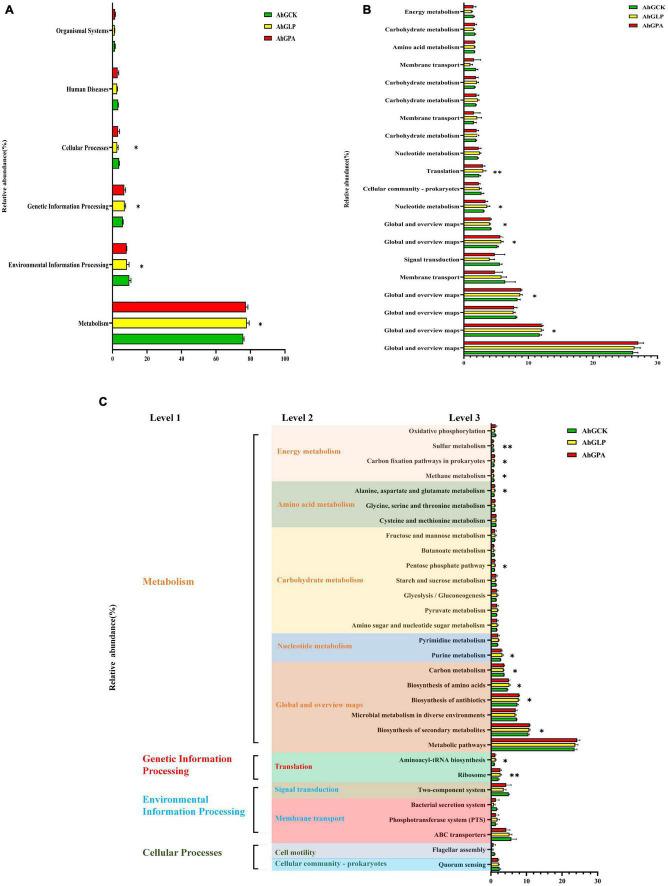
Functional predictions for silage microbiota with significantly different Kyoto encyclopedia of genes and genomes (KEGG) pathways (*p* < 0.05) among the three groups of silage (AhGCK, AhGLP, and AhGPA). KEGG pathways at level 1 **(A)**, level 2 **(B)**, and level 3 **(C)** are represented. AhGCK, control; AhGLP, *L. plantarum*; AhGPA, propionic acid. *p < 0.05; **p < 0.01.

Kyoto encyclopedia of genes and genomes (KEGG) is a bioinformatics tool for understanding the capacities and utilities of cells and living beings at both high-level and genomic points of view. The prediction of useful shifts in bacterial communities facilitates the assessment of the impact of organisms on energy changes in silage quality. KEGG pathway analysis was therefore used to predict the function of the microbial community of high-moisture amaranth silage. A total of 364 predicted functions were generated, some of which were significantly different between the three silage groups (Figure 5). The main predicted functional genes at level 1 during fermentation were distributed among the functions of metabolism, environmental information processing, genetic information processing, and cellular processes (Figure 5A). Compared with the control (AhGCK), metabolism and genetic information processing were significantly increased in AhGLP and AhGPA, while the functions of environmental information processing and cellular processes were significantly reduced. This may be because the growth of LAB in silage without additives is much lower compared with that in the additive silage, thus the growth and metabolic activity of other microorganisms is inhibited, along with the biosynthesis of secondary metabolites and antibiotics. KEGG pathway levels 2 and 3 of bacterial gene function are shown in Figures 5B, C. In the metabolism function, there were significant differences or highly significant differences between the control and additive-treated silage (*p* < 0.05 or *p* < 0.01) in the KEGG pathway terms of biosynthesis of secondary metabolites; biosynthesis of antibiotics; biosynthesis of amino acids, carbon metabolism, purine metabolism, pentose phosphate pathway; alanine, aspartate, and glutamate metabolism; methane metabolism; carbon fixation pathways in prokaryotes; and sulfur metabolism. In contrast, aminoacyl-tRNA biosynthesis and ribosome in the genetic information processing function showed significant or highly significant differences (*p* < 0.05 or *p* < 0.01) between the control and additive-treated silage. Sulfur metabolism, methane metabolism, and carbon metabolism were lower in the additive groups compared with the control group. All KEGG pathways at level 3 were enriched in the silage containing additives compared with the control. The specific fermentation characteristics of the different silage groups showed that the exogenous microbiota of amaranth improved the quality of the fermentation by altering the cellular characteristics, inhibiting membrane transport and signal transduction of undesirable bacteria, and accelerating the rate of multiplication and metabolic levels of beneficial bacteria such as LAB species.

The fermentation process of silage comprises microbial activity to degrade substrates or convert metabolites through complex metabolic pathways. The additives facilitated the metabolic pathways of carbohydrates in silage in this study, which was in line with the metabolic pathways associated with silage fermentation that were identified by Xu et al. (2021)—namely, the metabolism of carbohydrates, amino acids, energy and cofactors, and vitamins. This indicates that silage microorganisms (*Lactiplantibacillus plantarum* and *Lentilactobacillus buchneri*) complemented with *L. plantarum* and PA had a greater ability to metabolize WSC. The expression of carbohydrate metabolic pathways was correlated with the relative abundance of total LAB in the bacterial community. A relatively high abundance of total LAB and a poor relative abundance of carbohydrate metabolism in the bacterial community was observed in AhGLP. According to Pessione et al. (2010), the three main energy metabolism pathways in *Lactobacillus* were amino acid decarboxylation, malate decarboxylation, and arginine IX decarboxylation, and these pathways contributed to the accumulation of LA during fermentation. This differed from our study, possibly due to the low carbohydrate content of amaranth and its high consumption as a fermentation substrate during the silage process. Homofermentative LAB have been classified as obligate homofermentative and facultative heterofermentative (Orla-Jensen, 1919; Kandler, 1983; Axelsson, 2004). Obligate homofermentative LAB mainly metabolize hexose through the glycolytic pathway and then convert it all to pyruvate, which is reduced to lactic acid by lactate dehydrogenase. Facultative heterofermentative LAB metabolize pentose *via* the pentose phosphate pathway to produce LA and AA. Interestingly, obligate homofermentative LAB do not metabolize pentose or gluconate. The carbohydrate metabolism pathway of level 3 suggested that the *L. plantarum* added to our study may belong to the facultative heterofermentative. Silages with different additives showed variability in the pentose phosphate pathway, with some heterogeneously fermented LAB producing AA *via* this pathway. This indirectly explains the higher relative abundance of *Lactilantibacillus* and *Lentilactobacillus* in AhGLP. At the same time, this suggests that AhGLP has more heterotypic fermentation processes and a richer metabolic pathway than AhGCK and AhGPA. Furthermore, the epiphytic *Lactiplantibacillus plantarum* and *Lentilactobacillus buchneri* could inhibit the reproductive activity of undesirable microorganisms to a certain extent, thus slowing the spoilage process of the silage.

Amino acids are the basic building blocks of proteins. While the highest CP content was found in silage inoculated with PA, the highest metabolic abundance of alanine, aspartate, and glutamate metabolism was found in silage inoculated with *L. plantarum*. This was consistent with the lowest pH and NH_3_-N detected in silage inoculated with *L. plantarum.* The relative abundance of *X. oryzae* was higher in AhGPA compared with that in AhGCK and AhGLP, which may explain why AhGPA had the highest CP content but differed from the silage supplemented with *L. plantarum* in terms of metabolic rate and silage quality. The low metabolism might reflect the capacity of the initial microbial populations, in the silage, to synthesize amino acid *de novo*. In contrast, as LAB do not synthesize all their essential amino acid, they rely on proteolytic systems to provide essential amino acids for their growth (Novik and Savic, 2020). Therefore, the dynamics of amino acid metabolism observed in AhGCK and additive-treated silages may reflect the metabolism of the dominant populations throughout the ensiling process. Above all, the low pH of silage inoculated with *L. plantarum* inhibited amino acid metabolism induced by the undesirable microorganisms of the silage such as *Enterobacter cloacae* and *X. oryzae* (Flythe and Russell, 2004; Yuan et al., 2020). Despite these observations, the reliability of KEGG gene function predictions is limited, and we will therefore combine the KEGG analysis with metabolomics for multi-omics validation in future work.

## Conclusion

Inoculation of the epiphytic microbiota with *L. plantarum* and PA significantly altered the fermentation products and bacterial community composition and predicted metabolic pathways of high-moisture amaranth silage. The dominant bacterial species of AhGLP were *Lactiplantibacillus plantarum*, *Enterobacter cloacae*, and *Lentilactobacillus buchneri*. The predominant bacterial species of AhGLP were *Lactiplantibacillus plantarum*, *Enterobacter cloacae*, and *X. oryzae*. LAB dominated both additive silages. Furthermore, *L. plantarum* markedly inhibited the reproductive activity of *X. oryzae* and reduced the depletion of nutrients despite amaranth being a known host of phytopathogenic *X. oryzae*. However, the effect of exogenous PA on the bacterial community of the silage was limited. In summary, our results confirm the feasibility of regulating high-moisture amaranth silage by inoculation with *L. plantarum*.

## Data availability statement

The datasets presented in this study can be found in online repositories. The names of the repository/repositories and accession number(s) can be found below: https://www.ncbi.nlm.nih.gov/, PRJNA888204.

## Author contributions

MZ: conceptualization, investigation, visualization, methodology, formal analysis, and writing—original draft. ZW: supervision and writing—review and editing. SD, LS, JB, and JH: conceptualization, investigation, and formal analysis. GG: conceptualization, methodology, validation, investigation, writing—review and editing, and funding acquisition. All authors contributed to the article and approved the submitted version.

## Footnotes

1
https://international.biocloud.net
